# The courts as political players

**DOI:** 10.1186/s13584-019-0300-1

**Published:** 2019-03-18

**Authors:** Sara Rosenbaum

**Affiliations:** Department of Health Policy and Management, Milken Institute School of Public Health, George Washington University, 2175 K. Street N.W. Suite 500, Washington D.C., 20037 USA

**Keywords:** Courts, Judicial review, Health reform, Health governance and law

## Abstract

Courts are political animals; as a result, they strive not only to carry out their assigned role in national governance but also to balance that role within the political and social system in which they operate. Sperling and Cohen offer an elegant, in-depth analysis of how these institutional interests have helped shape decision-making by the Israeli Supreme Court in a health policy context. In doing so, the authors tell a universal story, one with enormous resonance in the U.S.

## Main text

If asked whether courts are political animals, any lawyer – probably anywhere in the world -- with any feel for the judicial system in which he or she operates likely would smile at the question. Of course they are, would be the answer. It is not just a matter of how judges are selected – which, depending on the system, might be through periodic election or confirmation to lifetime appointment.^1^ The political life of courts begins with judges themselves. To be sure, there are judges like United States Supreme Court Justice Sonia Sotomayor, who have overcome astounding odds to serve at the highest level. But in the United States, most judges, especially those at the appellate level, possess privileged backgrounds; they have attended the most elite schools, clerked for the most elite courts, and pursued the most elite careers in public or private law. Inevitably such judges bring a certain world view both on the bench and in society [2]. Furthermore, the selection process is intensely political, as Linda Greenhouse, the New York Times’ celebrated former Supreme Court reporter and long-time observer of the Court reminded Americans in the wake of the extraordinary Kavanaugh hearing spectacle that riveted the nation in the fall of 2018. Here, one need look no further than the Federalist Society (a private judicial lobbying group) and its impact on the federal judicial appointment process under the Trump Administration to understand the link between the courts and politics. [2].

### The political life of courts

But the political life of courts goes beyond judicial selection and extends to the judicial process as well. Here in the U.S. politics show up in the blizzard of procedural rules, many grounded in tradition, regarding not only which cases get heard but decision-making itself. These rules, conventions, and traditions can be stretched, bent, and restated in ways that fundamentally influence what cases qualify for judicial review and which get decided on preliminary procedural grounds or reach the actual merits. This is particularly true with the highest reviewing court in any nation’s judicial system, where each move – which cases are accepted for review and which are not,^2^ what questions the reviewing court decides to consider, what is said during oral arguments – is intently watched and analyzed, sometimes to the point of absurdity.

### The Sperling and Cohen analysis

Here in the U.S. we currently are in the midst of one of these major “reading the judicial tea leaves” moments in health policy. This case possesses all the dramatic elements of those examined by Daniel Sperling and Nissim Cohen in their penetrating analysis of the politics of Israel’s Supreme Court in the context of health care policy. Through elegant, clear, and informative writing – even for readers such as myself who have the barest familiarity with the Israeli legal system -- the authors demonstrate how a court’s institutional interest in its place in society ultimately can shape the cases it hears and whether it will focus on preliminary aspects or reach the heart of the matter, in this article, the existence and extent of health care rights conferred on individuals under law.

To illustrate their main thesis – that the Court cares deeply about its place within a democratic society, and thus its political place in the policymaking process -- the authors examine several important, recent decisions that potentially raise the deepest questions regarding health care rights under law. In so doing, the authors offer important insight into how the modern Israeli Supreme Court balances its position within a democracy against its role in protecting individuals against administrative and legislative overreach. The authors demonstrate how the Court has constrained the reach of its decisions in order to avoid interfering with the democratic process, while at the same time recognizing its protective obligations. The results may be decisions that appear to favor certain philosophical views about how to balance individual interests against broader political and social currents. But as the authors note, the Court is guided most deeply by recognition of its position in the national scheme of governance rather than its power to answer ultimate questions regarding legal rights.^3^

### The analysis in a U.S. judicial context

Courts in the United States face this judicial dilemma all the time, most spectacularly at the moment in a case involving health policy, as well. In December 2018, in *Texas v United States,* a single federal judge sitting in Fort Worth, Texas, acting against all rational legal reasoning, ruled unconstitutional the nation’s entire 2010 health reform law, the Patient Protection and Affordable Care Act (ACA). Health insurance coverage for over 20 million people is at stake, as are all of the law’s other reforms, including comprehensive insurance regulation, changes in health care service delivery and payment, and public health revisions.

Beyond the initial shock of the decision have come a large number of questions; ultimately, the question is what does the Supreme Court do with such a hot mess? The Court already has twice saved the ACA from legal oblivion through two decisions [3], *National Federation of Independent Businesses v Sebelius* and *King v Burwell*. Both cases involved sustained efforts to weaponize the courts in order to bring down a law that has proven legislatively unstoppable [1]. The first case, like the current one, involved the law’s basic constitutionality; the second focused the law’s critical insurance subsidy provisions, designed to make coverage affordable for millions of individual purchasers.

As this newest onslaught makes its way through the judicial process, questions abound. Will it even reach the Court or will the intermediate appellate court -- itself one of our most conservative regional federal appellate courts -- dispose of this mess, either by overturning the decision or dismissing it on procedural grounds (of which there potentially are several)? Will the Supreme Court decide to review given the magnitude of the issue and regardless of the decision reached at the intermediate level? If so, will the Court once again save the ACA, either on procedural grounds or by reaching the ultimate merits of the case? In so doing, our Court, as apparently is the case with the Israeli Supreme Court, effectively will be telling litigants – 20 states still smitten with anti-ACA fervor, despite mid-term elections that reinforced the primacy of health care for American voters -- that the judicial process is not the place where the deepest health care politics get resolved.

## Conclusions

In nations in which the judiciary is given supreme authority over law, it nonetheless remains the case that judges will be cognizant of the political and social environments in which they operate. In the end, the responsible exercise of judicial power inevitably is an effort to balance the purpose and meaning of law against the real-world circumstances in interpretations of law will be applied. Good judging means recognizing relationship between judicial authority on one hand and politics, and society on the other. Indeed, it is this recognition of the importance, whenever possible, of considering how judicial interpretation will affect people and the broader social order that gives the judicial process its power and helps assure the public that the judicial system is not an exercise in authoritarianism but is, instead, indispensable to the fabric of life.

## Abbreviations

**ACA**: **Patient Protection and Affordable Care Act (commonly referred to as the Affordable Care Act) – national health reform legislation enacted by the United States Congress and signed into law by President Obama in 2010, which is considered the most significant single piece of health reform legislation in the U.S. since the enactment of Medicare and Medicaid in 1965. The legislation introduced major market reforms to private health insurance sold both to individuals and group. Most notably are: a ban on refusing to sell or renew policies based on health status; a ban on pre-existing condition exclusion clauses; a ban on pricing discrimination; a requirement to use modified community rating in pricing health insurance; and minimum coverage for “essential health benefits” coupled with minimum medical/loss ratios; and limits on out-of-pocket financial exposure for policies subject to the law’s market rules. The tax penalty for failing to purchase adequate insurance coverage, part of the law on enactment, was reduced to zero by tax legislation enacted in 2017. The ACA also expanded Medicaid to coverage of the lowest income working age adults ineligible under traditional program rules, established a system of premium subsidies to make coverage affordable for low and moderate income individuals and families who rely on the individual insurance market rather than employer coverage or public insurance, established health insurance Exchanges (known as Marketplaces) for the sale of affordable individual policies, and contained major Medicare coverage and payment reforms. The ACA contained numerous other reforms to key public health programs, nonprofit tax policy, and made changes in public health regulatory systems.**

## Footnotes

1 Israel makes a more explicit effort to minimize politics in judicial selection. By law, the appointment of judges in Israel is based on experience, skills, integrity and demeanor. Unlike the U.S., judges are chosen by a nine-member committee that attempts to balance politics with abilities; the nine-member special committee includes both politicians and others. At least four must be women, and membership consists of two members of the Knesset, two government ministers, three Justices of the Supreme Court and two representatives of the Israel Bar Association. While there is a political dimension to the appointments even in Israel, Israel makes an active effort to keep politics to a limit.

2 Unlike the rarest of circumstances in the U.S., and perhaps due in part to Israel’s far smaller size, the Israeli Supreme Court usually becomes the first voice in constitutional challenges. In Israel, constitutional challenges to primary legislation are generally brought directly to the Supreme Court, which cannot decide not to hear the case. In Israel, each year the Supreme Court hears thousands of cases in which it gives a reasoned verdict. By contrast, the U.S. Supreme Court hears fewer than 100 such cases each year and can decide to allow a lower court challenge to stand unreviewed, although such a decision would be unusual to say the least.

3 Indeed, the Israeli Supreme Court operates in a political environment. The political bodies, chiefly the Knesset, can, at least theoretically, change the “rules of the game” and limit the jurisdiction of the courts (or threaten to do so). It is easier to change Basic Laws in Israel than to amend the Constitution in the United States [4]. The court cannot disengage itself from the delicate relations among the branches of government and it exercises restraint in deciding when and how to intervene in administration policy and the legislation of the Knesset.
